# Association Between Serum Folate Concentrations and 10-Year Stroke Risk in a Prospective Community Cohort: Mediation and Interaction Analyses

**DOI:** 10.3390/nu17010159

**Published:** 2024-12-31

**Authors:** Zhe Liang, Fangfang Fan, Bo Liu, Kaiyin Li, Hongyu Chen, Jia Jia, Yong Huo, Jianping Li, Yan Zhang

**Affiliations:** 1 Department of Cardiology, Peking University First Hospital, No. 8 Xishiku Street, Xicheng District, Beijing 100034, China; liangzhechn@163.com (Z.L.); fang9020@126.com (F.F.); liubo3077@163.com (B.L.); kaiyin0313@foxmail.com (K.L.); chy7449@163.com (H.C.); jiajia9985@163.com (J.J.); huoyong@263.net.cn (Y.H.); lijianping03455@pkufh.com (J.L.); 2 Institute of Cardiovascular Disease, Peking University First Hospital, No. 8 Xishiku Street, Xicheng District, Beijing 100034, China

**Keywords:** folate, homocysteine, *MTHFR C677T* gene polymorphism, stroke, cohort study, epidemiology

## Abstract

The relationship between folate concentrations and stroke risk remains unestablished, and the mediation effect of homocysteine (Hcy) and interaction effect of methylenetetrahydrofolate reductase (*MTHFR*) *C677T* gene polymorphism has yet to be investigated. This cohort study involved 4903 subjects derived from a Chinese community population. The association between folate and first stroke was examined in Cox proportional hazard regression models. The mediation analyses involving Hcy and the undiscovered modification of the *MTHFR C677T* genotype were assessed. The mean (SD) age of subjects was 56.7 (8.8) years old, and 37.0% were male. A total of 407 strokes, 375 ischemic strokes and 47 hemorrhagic strokes occurred during the mean (SD) follow-up of 9.3 (1.8) years. The participants in the highest folate quartile (≥8.2 ng/mL) exhibited a lower stroke risk compared to those in the lowest quartile (hazard ratio [HR]: 0.68, 95% confidence interval [CI]: 0.50–0.93, *p* = 0.017; *p* for trend = 0.009). Hcy significantly mediated 14.51% of the relationship between folate and stroke in the fully adjusted model. Only in individuals with the *MTHFR 677CC* genotype but not *CT*/*TT* genotype was folate adversely correlated with stroke (HR: 0.88, 95% CI: 0.79–0.97, *p* for interaction = 0.026) and ischemic stroke (HR: 0.88, 95% CI: 0.80–0.98, *p* for interaction = 0.035). Insufficient folate concentrations were associated with a heightened 10-year stroke risk, in which Hcy concentrations played a crucial mediating role. *MTHFR C677T* gene polymorphism could potentially modify the folate–stroke relationship.

## 1. Introduction

Folate is a vital water-soluble micronutrient, and includes naturally occurring folate in the body as well as synthetic folic acid in supplements and fortified foods [1]. Folate plays a crucial role in cell development and differentiation, DNA and histone methylation in epigenetics, and the regulation of immune cell functions [2]. Current epidemiologic studies have identified an association of inadequate folate concentrations with elevated all-cause and cardiovascular mortality [3,4,5,6].

Given its involvement in one-carbon metabolism, specifically concerning homocysteine (Hcy) [7], which is recognized by the American Heart Association to be associated with stroke and other vascular diseases [8], it was hypothesized that folic acid intake or plasma/serum folate concentrations may affect stroke risk by modulating Hcy concentrations. The recent systematic review of randomized controlled intervention trials has demonstrated that adequate folic acid supplementation could reduce stroke risk [9]. However, the evidence concerning the association between folate concentrations and stroke risk remains scarce and contradictory, especially across different stroke types. One nested case-referent study suggested that folate might have a protective effect on hemorrhagic rather than ischemic stroke [10]. A prospective population-based study found no significant association between plasma folate concentrations and ischemic stroke risk, whereas dietary folic acid was negatively correlated with ischemic stroke [11].

Methylenetetrahydrofolate reductase (MTHFR) is an essential enzyme in folate and Hcy metabolism. It facilitates the irreversible conversion of 5,10-methylenetetrahydrofolate into 5-methylenetetrahydrofolate, a circulating form of folate involved in the remethylation of Hcy to methionine [12]. *MTHFR C677T* polymorphism (rs1801133) disrupts methylation capacity, activates inflammatory responses, induces lipid metabolism disorders, and promotes endothelial dysfunction and thrombosis, serving as a representative of constant exposure to low folate and high Hcy concentrations [13]. Either a whole-genome sequencing analysis or another prospective study uncovered a link between the *C677T* mutation and elevated stroke risk in the low-folate Chinese population [14,15]. A recent meta-analysis of case–control studies revealed that the T allele of *MTHFR C677T* might be a risk factor for ischemic stroke [16].

To date, the roles of folate concentrations in stroke remain unestablished, and the impact of Hcy and potential modification of the *MTHFR C677T* gene polymorphism on the folate–stroke relationship in the general population have yet to be investigated. The current study aims to analyze the relationship between serum folate concentrations and a 10-year risk of stroke among a Chinese community cohort without a history of cardiovascular disease (CVD). Importantly, the mediation effect of Hcy and potential inheritance–metabolism interaction on stroke will also be investigated.

## 2. Materials and Methods

### 2.1. Study Design and Population

The participants were recruited from an atherosclerosis cohort in two communities in Shijingshan District, Beijing, China. A total of 9540 individuals aged 40 and above were initially enrolled in the baseline survey conducted between December 2011 and April 2012. A 10-year follow-up survey was then carried out from 2011 to 2021. Participants with missing follow-up information, lacking serum folate and plasma Hcy concentrations, ineligible *MTHFR C677T* genotype and a history of baseline CVD, defined as self-reported myocardial infarction or stroke (including transient ischemic attack), were excluded. A total of 4903 subjects were included for subsequent data analysis (Figure S1). This study adhered to the Strengthening the Reporting of Observational Studies in Epidemiology (STROBE) guidelines [17].

### 2.2. Demographic and Clinical Characteristics

Demographic data, such as baseline age, sex, lifestyle, and medical history, were gathered by trained healthcare practitioners. Body mass index (BMI) was computed as dividing weight in kilograms by the square of height in meters. In-depth interviews were conducted about alcohol consumption (drinking once or more weekly for over six months) and tobacco usage (smoking at least one cigarette daily for over six months). Blood pressure was measured by an electronic sphygmomanometer (Omron HEM-7117, Omron, Kyoto, Japan). Hypertension was defined as systolic blood pressure ≥ 140 mmHg, diastolic blood pressure ≥ 90 mmHg, self-reported history or usage of antihypertensive medication. Dyslipidemia was identified by low-density lipoprotein cholesterol ≥ 3.4 mmol/L, high-density lipoprotein cholesterol < 1.0 mmol/L, triglycerides ≥ 1.7 mmol/L, total cholesterol ≥ 5.2 mmol/L, self-reported history or usage of lipid-lowering medication. Diabetes was characterized by fasting blood glucose level ≥ 7 mmol/L, postprandial blood glucose level ≥ 11.1 mmol/L, self-reported history or usage of antidiabetic medication.

### 2.3. Determination for Biochemical Variables

Serum concentrations of glucose, lipids and creatinine were measured by the Roche C8000 Automatic Biochemical Analyzer (Roche, Basel, Switzerland). The estimated glomerular filtration rate (eGFR) was calculated based on the Chronic Kidney Disease Epidemiology Collaboration equation [18], and abnormal renal function was defined as eGFR below 90 mL/min/1.73 m^2^. Employing the Maglumi4000 automated chemiluminescent immunoassay analyzer (SNIBE, Shenzen, China), serum folate concentrations were measured by the electrochemiluminescence method. Utilizing the Beckman Coulter AU480 automated biochemical analyzer (Beckman Coulter, Brea, CA, USA), plasma Hcy concentrations were determined through the enzymatic cycling method. The *MTHFR C677T* genotype was tested using the Asian ExomeChip, a custom exome array built upon the Infinium HumanExome BeadChip (Illumina, San Diego, CA, USA), as previously documented [19].

### 2.4. Ascertainment of Outcomes

The Chinese Center for Disease Control and Prevention and the Beijing Municipal Health Commission, through their National Mortality Surveillance System and Inpatient Medical Record Home Page System, respectively, contained stroke information for all participants until the occurrence of events or completion of follow-up (31 December 2021). Events were categorized by the International Classification of Diseases, 10th Revision (ICD-10). The primary endpoint was the first stroke (fatal and non-fatal), comprising ischemic stroke (I63), hemorrhagic stroke (I60–I61) and other types of stroke (I64). The secondary endpoints were the first ischemic stroke (I63) and the first hemorrhagic stroke (I60-I61).

### 2.5. Statistical Analysis

We employed descriptive statistics to outline the characteristics of the entire cohort and by quartiles of serum folate concentrations. Mean (standard deviation, SD) or median (interquartile range, IQR) were computed for continuous variables, while frequencies were tabulated for categorical variables. Baseline differences across folate quartiles were assessed using a one-way analysis of variance alongside the Kruskal–Wallis test for continuous variables and chi-squared analysis for categorical variables, accordingly. The Kaplan–Meier method was employed to assess the cumulative stroke hazards stratified by folate concentrations, with group differences determined through log-rank tests. The association between folate concentrations and endpoints was explored in Cox proportional hazard regression models, adjusting for various covariates such as baseline age, sex, BMI, eGFR, drinking, smoking, hypertension, dyslipidemia, diabetes and the use of antihypertensive, lipid-lowering and hypoglycemic drugs in Model 1, with further adjustment for Hcy concentrations in Model 2.

We conducted mediation analyses utilizing Hcy to evaluate both the direct and indirect relationship between folate and stroke. The mediated proportion of Hcy was computed: mediation effect (%) = (β_a_ × β_b_/β_Total_) × 100% (β_a_ = indirect effect1, β_b_ = indirect effect2, β_Total_ = total effect). This methodology has been previously reported on and employed [20,21]. Based on stratified analyses and interaction tests, we examined the potential modification of *MTHFR C677T* gene polymorphism on the association between folate concentrations and stroke. Restricted cubic spline (RCS) analyses after full adjustment were used to assess the dose–response relationship between folate and stroke in the overall population, as well as in the subgroups stratified by *MTHFR C677T* genotype.

A two-sided *p* value < 0.05 was regarded as statistically significant. All analyses were completed via R 4.3.3 (http://www.R-project.org, accessed on 15 September 2024).

## 3. Results

### 3.1. Baseline Characteristics

Baseline characteristics of the final cohort and different subsets stratified by quartiles of folate concentrations are presented in Table 1. The mean (SD) age of the 4903 subjects was 56.7 (8.8) years old, and 37.0% (n = 1815) were male. The median (IQR) values of serum folate and plasma Hcy were 6.2 (5.0, 8.2) ng/mL and 11.9 (10.0, 14.8) μmol/L. The distribution of the *MTHFR C677T* genotype (*CC*, *CT* and *TT*) within the total cohort was 18.7% (n = 915), 46.8% (n = 2295) and 34.5% (n = 1693), respectively. The median (IQR) values of serum folate across various quartiles were 4.4 (4.0, 4.7) ng/mL in Q1, 5.5 (5.2, 5.8) ng/mL in Q2, 7.0 (6.6, 7.6) ng/mL in Q3 and 10.3 (9.1, 12.3) ng/mL in Q4. As serum folate concentrations reduced, it was logical to observe an increase in plasma Hcy concentrations, along with a gradient decrease in the proportion of the *MTHFR 677CC* genotype.

### 3.2. Primary and Secondary Endpoints

**Figure 1:**
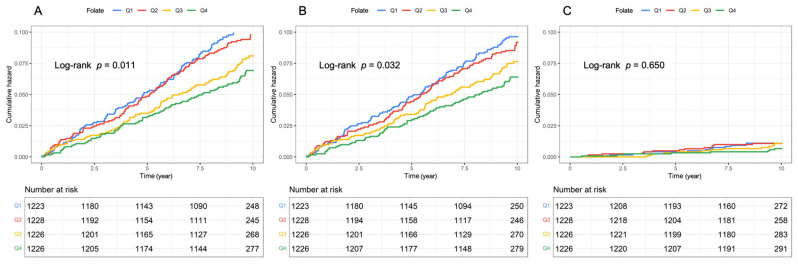
Kaplan–Meier survival curves of the cumulative stroke hazards. (**A**) Stratified by quartiles of serum folate concentrations in stroke; (**B**) stratified by quartiles of serum folate concentrations in ischemic stroke; (**C**) stratified by quartiles of serum folate concentrations in hemorrhagic stroke.

A total of 407 strokes, 375 ischemic strokes and 47 hemorrhagic strokes occurred during the mean (SD) follow-up of 9.3 (1.8) years. The Kaplan–Meier survival curves (Figure 1) showed a significant visual relationship of folate concentrations with the 10-year incidence of stroke and ischemic stroke (log-rank tests *p* < 0.05 for both) but no significant cumulative hazard for hemorrhagic stroke.

**Figure 2:**
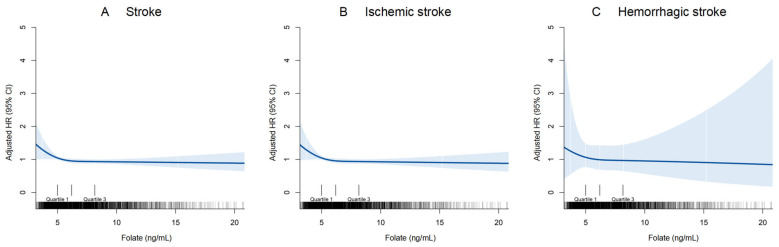
Restricted cubic spline of the dose–response relationship between serum folate concentrations and stroke. Restricted cubic spline was adjusted for baseline age, sex, BMI, eGFR, drinking, smoking, hypertension, dyslipidemia, diabetes, the use of antihypertensive, lipid-lowering and hypoglycemic drugs and plasma Hcy concentrations. The blue shaded region denotes the 95% CI for the restricted cubic spline. The X-axis is bounded by the 0.5th and 99.5th percentiles of serum folate concentrations. CI: confidence interval; HR: hazard ratio.

The unadjusted and adjusted Cox regression analyses of folate concentrations for stroke are shown in Table 2. In terms of the primary endpoint, participants in the highest quartile of serum folate concentrations (≥8.2 ng/mL) exhibited a significantly lower risk of stroke compared with those in the lowest quartile (an absolute risk reduction: 3.3%; hazard ratio [HR]: 0.64, 95% confidence interval [CI]: 0.47–0.87, *p* = 0.005; *p* for trend = 0.002) after adjustment for multiple covariates in Model 1. Following additional adjustments for plasma Hcy concentrations in Model 2, the association between folate and stroke risk still existed (HR: 0.68, 95% CI: 0.50–0.93, *p* = 0.017; *p* for trend = 0.009). As for secondary endpoints, participants in the highest quartile of folate had a 36% reduced risk of ischemic stroke compared to those in the lowest quartile of folate (HR: 0.64, 95% CI: 0.47–0.88, *p* = 0.007; *p* for trend = 0.005). The results also remained stable after further adjustment for Hcy. Folate concentrations, whether as a continuous or categorical variable, showed no significant association with the risk of hemorrhagic stroke. The results of RCS analyses revealed that, after adjusting for covariates, the risk of stroke and ischemic stroke decreased initially and then leveled off as serum folate concentrations increased (Figure 2).

### 3.3. Mediation Analyses

**Figure 3:**
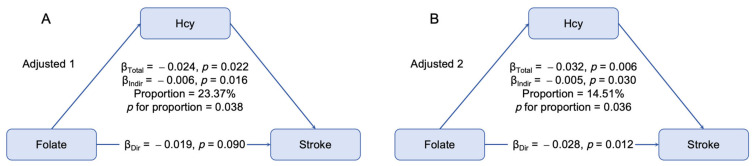
Mediation effect of plasma Hcy concentrations on the association between serum folate concentrations and stroke. (**A**) Model 1 was adjusted for baseline age and sex. (**B**) Model 2 was adjusted for the covariates in model 1 plus BMI, eGFR, drinking, smoking, hypertension, dyslipidemia, diabetes and the use of antihypertensive, lipid-lowering and hypoglycemic drugs. Hcy: homocysteine.

Based on the persistent association between folate and stroke risk after adjusting for Hcy, we further explored the mediation effect of Hcy (Figure 3). With adjustments for baseline age and sex alone, the mediation effect of Hcy accounted for 23.37%. In the fully adjusted model, the direct effect of folate and indirect effect of Hcy on stroke were −0.028 (95% CI: −0.048–−0.005, *p* = 0.012) and −0.005 (95% CI: −0.009–−0.001, *p* = 0.030), respectively. Hcy partially mediated the relationship between folate and stroke risk, with a mediated proportion of 14.51% (*p* for proportion = 0.036).

### 3.4. Stratification Analyses

We conducted stratified analyses to investigate how *MTHFR C677T* gene polymorphism might modify the relationship between folate, treated as a continuous variable, and stroke risk. In individuals with the *CC* genotype, elevated serum folate concentrations were significantly associated with a decreased risk of stroke (HR: 0.88, 95% CI: 0.79–0.97, *p* for interaction = 0.026) and ischemic stroke (HR: 0.88, 95% CI: 0.80–0.98, *p* for interaction = 0.035), but not with the risk of hemorrhagic stroke (Table 3). RCS analyses following stratification by the *MTHFR C677T* genotype are demonstrated in Figure S2. The smooth curve for the *CC* genotype indicated a more notable reduction in the risk of stroke and ischemic stroke with folate concentrations rising compared with the smooth curve for the *CT*/*TT* genotype.

## 4. Discussion

To our knowledge, this is the first prospective large-scale cohort study utilizing mediation analysis to delve into the role of plasma Hcy concentrations and explore the interaction effect of the *MTHFR C677T* genotype in the association between folate concentrations and stroke through long-term follow-up in the general population. We confirmed that insufficient folate concentrations were linked to a heightened 10-year stroke risk, which was significantly mediated by plasma Hcy concentrations. In addition, the negative correlation between folate and the risk of stroke was solely observed in individuals with the *CC* genotype.

Folic acid supplementation has been demonstrated to mitigate stroke risk by diminishing oxidative stress, increasing brain-derived neurotrophic factors, promoting angiogenesis and facilitating cellular adaptation to ischemia and hypoxia [22,23]. The results of the China Stroke Primary Prevention Trial (CSPPT), conducted by our team, indicated that supplementation of folic acid combined with antihypertensive medication could reduce the risk of first stroke in hypertensive individuals by up to 21% compared to antihypertensive treatment alone [24]. The protective effect of folic acid on stroke may reflect a combined impact of various protective nutrients or dietary elements in one-carbon metabolism, while serum folate accurately reflects the body’s folate status. In our study, elevated folate concentrations were negatively associated with a ~10-year stroke risk. One prospective cohort study showed that elevated serum folate concentrations were associated with a decreased risk of all stroke and ischemic stroke in Eastern Finnish middle-aged men [25]. A Mendelian randomization study also revealed that higher genetically predicted folate concentrations were linked to a reduced stroke risk [26], which verifies that the relationship between them is consistent and solid in both East Asian and European populations.

This is the first study to quantify the mediation effect of Hcy in the folate–stroke relationship. A cohort study hypothesized that the association between combined low folate/vitamin B_12_ concentrations and an increased cerebrovascular ischemia risk was partially mediated by Hcy metabolism [27]. Another population study indicated that Hcy mediated the relationship between vitamin B_12_ and various diseases with small effects [28]. Elevated Hcy concentrations may increase the stroke risk by raising blood pressure, causing endothelial dysfunction, releasing vasoactive substances and inducing vascular injury [29,30,31,32]. Folate has a direct effect on stroke, possibly due to the involvement of multiple metabolites biosynthesis, epigenetics and redox homeostasis in the one-carbon metabolism. Further research is needed to explore the pathophysiological mechanisms by which folate influences stroke regardless of Hcy.

Innovatively, this study found that *MTHFR C677T* gene polymorphism could significantly modify the folate–stroke relationship, with the negative association being more pronounced in individuals with the *CC* genotype. Similarly, the CSPPT study revealed that among the hypertensive population with the *CC*/*CT* genotype, a negative correlation was observed between baseline folate concentrations and stroke risk. In contrast, individuals with the *TT* genotype consistently exhibited a high stroke risk across all folate concentrations [24]. Another post hoc analysis demonstrated that compared to participants with the *CT*/*TT* genotype, elevated concentrations of folate or vitamin B_12_ were notably associated with a reduced risk of first ischemic stroke among hypertensive patients with the *CC* genotype [33]. The *MTHFR 677C* → *T* mutation reduces efficiency in folate and Hcy metabolism. Therefore, exploring the relationship between 5-methyltetrahydrofolate concentrations and stroke risk needs to be further conducted for individuals with the insensitive *CT*/*TT* genotype, as 5-methyltetrahydrofolate can bypass the interference of low MTHFR activity on folate concentrations [34].

This study also has some limitations. Firstly, serum folate concentrations were assessed at the baseline, which might not precisely represent their long-term fluctuations. Numerous studies have encountered similar limitations, and further investigation into the longitudinal impact of folate changes is warranted. Secondly, atrial fibrillation (AF) is known to be a major risk factor for ischemic stroke, especially embolic stroke. Due to the limited data on AF and type of ischemic stroke, we were unable to adjust for AF as a confounding factor in the relationship between folate and stroke, nor could we explore the association between folate and embolic stroke. Thirdly, due to the limited incidence of hemorrhagic stroke, conclusions should be interpreted with caution, necessitating further in-depth research. Lastly, since this study was conducted at a single center in northern China, our findings should be validated in other cohorts.

## 5. Conclusions

In summary, among the general Chinese population, we have discovered an association between elevated serum folate concentrations and a reduced ~10-year risk of stroke, particularly ischemic stroke. Hcy had a significant mediation effect on the folate–stroke relationship. Only in participants with the *MTHFR 677CC* genotype were serum folate concentrations adversely correlated with the risk of stroke and ischemic stroke. This study provides insights for further understanding the crucial role of Hcy and the *MTHFR C677T* polymorphism in the association between folate and stroke.

## Figures and Tables

**Table 1:** Baseline characteristics of the participants.

Characteristics	Overall	Quartiles of Serum Folate Concentrations				*p* Value
		Q1	Q2	Q3	Q4	
N	4903	1223	1228	1226	1226	
Age (year), mean (SD)	56.7 (8.8)	56.3 (9.1)	56.8 (9.2)	56.7 (8.6)	57.1 (8.3)	0.127
Sex, N (%)						<0.001
Male	1815 (37.0)	739 (60.4)	497 (40.5)	342 (27.9)	237 (19.3)	
Female	3088 (63.0)	484 (39.6)	731 (59.5)	884 (72.1)	989 (80.7)	
Serum folate (ng/mL), median (IQR)	6.2 (5.0, 8.2)	4.4 (4.0, 4.7)	5.5 (5.2, 5.8)	7.0 (6.6, 7.6)	10.3 (9.1, 12.3)	<0.001
Plasma Hcy (μmol/L), median (IQR)	11.9 (10.0, 14.8)	15.1 (12.2, 20.8)	12.2 (10.4, 14.7)	11.3 (9.7, 13.3)	10.3 (8.9, 12.1)	<0.001
MTHFR C677T, N (%)						<0.001
CC	915 (18.7)	141 (11.5)	220 (17.9)	235 (19.2)	319 (26.0)	
CT	2295 (46.8)	469 (38.3)	586 (47.7)	640 (52.2)	600 (48.9)	
TT	1693 (34.5)	613 (50.1)	422 (34.4)	351 (28.6)	307 (25.0)	
BMI (kg/m^2^), mean (SD)	26.1 (3.4)	26.3 (3.3)	26.2 (3.3)	26.0 (3.4)	25.8 (3.5)	0.002
eGFR (mL/min/1.73 m^2^), mean (SD)	94.8 (12.9)	94.1 (12.9)	94.5 (13.2)	94.9 (12.8)	95.6 (12.6)	0.023
eGFR classification (mL/min/1.73 m^2^), N (%)						0.005
≥90	3464 (70.7)	826 (67.6)	854 (69.6)	880 (71.8)	904 (73.8)	
<90	1436 (29.3)	396 (32.4)	373 (30.4)	346 (28.2)	321 (26.2)	
Current drinking, N (%)	1161 (23.7)	455 (37.2)	304 (24.8)	241 (19.7)	161 (13.1)	<0.001
Current smoking, N (%)	965 (19.7)	480 (39.2)	245 (20.0)	164 (13.4)	76 (6.2)	<0.001
Prevalence of disease, N (%)						
Hypertension	2421 (49.4)	588 (48.1)	603 (49.1)	617 (50.3)	613 (50.0)	0.684
Dyslipidemia	3501 (71.4)	825 (67.5)	862 (70.2)	895 (73.0)	919 (75.0)	<0.001
Diabetes	1156 (23.6)	223 (18.2)	247 (20.1)	302 (24.6)	384 (31.3)	<0.001
Medication, N (%)						
Antihypertensive drugs	1485 (30.5)	339 (27.9)	362 (29.7)	410 (33.6)	374 (30.7)	0.019
Lipid-lowering drugs	454 (9.4)	80 (6.6)	107 (8.8)	127 (10.5)	140 (11.5)	<0.001
Hypoglycemic drugs	482 (9.9)	78 (6.4)	85 (6.9)	133 (10.9)	186 (15.2)	<0.001
Endpoint, N (%)						
Stroke	407 (8.3)	121 (9.9)	112 (9.1)	93 (7.6)	81 (6.6)	0.014
Ischemic stroke	375 (7.6)	110 (9.0)	102 (8.3)	88 (7.2)	75 (6.1)	0.039
Hemorrhagic stroke	47 (1.0)	13 (1.1)	13 (1.1)	13 (1.1)	8 (0.7)	0.656

BMI: body mass index; eGFR: estimated glomerular filtration rate; Hcy: homocysteine; IQR: interquartile range; MTHFR: methylenetetrahydrofolate reductase; SD: standard deviation.

**Table 2:** Association between serum folate concentrations and the risk of stroke.

Endpoints	N	No. of Events, N (%)	Crude		Adjusted 1 *		Adjusted 2 ^†^	
			HR (95% CI)	*p* Value	HR (95% CI)	*p* Value	HR (95% CI)	*p* Value
Stroke								
Linear trend								
Folate	4903	407 (8.3)	1.00 (0.98, 1.02)	0.628	0.99 (0.97, 1.01)	0.376	0.99 (0.97, 1.01)	0.434
Folate quartiles								
Q1 (<5.0 ng/mL)	1223	121 (9.9)	ref		ref		ref	
Q2 (5.0–6.2 ng/mL)	1228	112 (9.1)	0.91 (0.71, 1.18)	0.496	0.92 (0.70, 1.19)	0.523	0.98 (0.75, 1.29)	0.892
Q3 (6.2–8.2 ng/mL)	1226	93 (7.6)	0.75 (0.57, 0.99)	0.039	0.76 (0.57, 1.01)	0.061	0.82 (0.61, 1.09)	0.174
Q4 (≥8.2 ng/mL)	1226	81 (6.6)	0.65 (0.49, 0.86)	0.003	0.64 (0.47, 0.87)	0.005	0.68 (0.50, 0.93)	0.017
*p* for trend	4903	407 (8.3)	<0.001		0.002		0.009	
Ischemic stroke								
Linear trend								
Folate	4903	375 (7.6)	1.00 (0.98, 1.01)	0.701	0.99 (0.97, 1.01)	0.415	0.99 (0.97, 1.01)	0.461
Folate quartiles								
Q1 (<5.0 ng/mL)	1223	110 (9.0)	ref		ref		ref	
Q2 (5.0–6.2 ng/mL)	1228	102 (8.3)	0.92 (0.70, 1.20)	0.520	0.91 (0.69, 1.21)	0.523	0.96 (0.72, 1.28)	0.791
Q3 (6.2–8.2 ng/mL)	1226	88 (7.2)	0.78 (0.59, 1.04)	0.089	0.79 (0.59, 1.06)	0.115	0.83 (0.62, 1.13)	0.236
Q4 (≥8.2 ng/mL)	1226	75 (6.1)	0.66 (0.49, 0.89)	0.006	0.64 (0.47, 0.88)	0.007	0.68 (0.49, 0.93)	0.018
*p* for trend	4903	375 (7.6)	0.003		0.005		0.012	
Hemorrhagic stroke								
Linear trend								
Folate	4903	47 (1.0)	0.95 (0.86, 1.06)	0.393	0.97 (0.87, 1.07)	0.540	0.98 (0.89, 1.08)	0.627
Folate quartiles								
Q1 (<5.0 ng/mL)	1223	13 (1.1)	ref		ref		ref	
Q2 (5.0–6.2 ng/mL)	1228	13 (1.1)	0.99 (0.46, 2.13)	0.972	1.03 (0.47, 2.26)	0.932	1.20 (0.53, 2.71)	0.665
Q3 (6.2–8.2 ng/mL)	1226	13 (1.1)	0.99 (0.46, 2.13)	0.972	1.05 (0.47, 2.34)	0.914	1.21 (0.52, 2.80)	0.655
Q4 (≥8.2 ng/mL)	1226	8 (0.7)	0.60 (0.25, 1.46)	0.262	0.69 (0.27, 1.77)	0.443	0.79 (0.30, 2.07)	0.632
*p* for trend	4903	47 (1.0)	0.308		0.510		0.693	

* Model 1: Adjusted for baseline age, sex, BMI, eGFR, drinking, smoking, hypertension, dyslipidemia, diabetes and the use of antihypertensive, lipid-lowering and hypoglycemic drugs. ^†^ Model 2: Adjusted for the covariates in model 1 plus plasma Hcy concentrations. CI: confidence interval; HR: hazard ratio.

**Table 3:** Modifying effect of *MTHFR C677T* genotype on the association between serum folate concentrations and stroke.

Subgroup	N	No. of Events, N (%)	HR (95% CI) *	*p* Value	*p* for Interaction
Stroke					
*MTHFR C677T*					0.026
*CC*	915	68 (7.4)	0.88 (0.79, 0.97)	0.012	
*CT*	2295	190 (8.3)	1.00 (0.99, 1.01)	0.640	
*TT*	1693	149 (8.8)	1.00 (0.96, 1.03)	0.792	
Ischemic stroke					
*MTHFR C677T*					0.035
*CC*	915	65 (7.1)	0.88 (0.80, 0.98)	0.016	
*CT*	2295	176 (7.7)	1.00 (0.99, 1.01)	0.666	
*TT*	1693	134 (7.9)	0.99 (0.95, 1.04)	0.727	
Hemorrhagic stroke					
*MTHFR C677T*					0.246
*CC*	915	5 (0.5)	0.72 (0.43, 1.20)	0.203	
*CT*	2295	19 (0.8)	0.94 (0.79, 1.13)	0.507	
*TT*	1693	23 (1.4)	1.01 (0.96, 1.06)	0.720	

* Adjusted for baseline age, sex, BMI, eGFR, drinking, smoking, hypertension, dyslipidemia, diabetes, the use of antihypertensive, lipid-lowering and hypoglycemic drugs and plasma Hcy concentrations. CI: confidence interval; HR: hazard ratio; MTHFR: methylenetetrahydrofolate reductase.

## Data Availability

The corresponding authors had complete access to all the study data and are accountable for its integrity and the data analysis. The datasets analyzed in this study are available from the corresponding authors upon reasonable request. The data are not publicly available due to being part of an ongoing study.

## Supplementary Materials

The following supporting information can be downloaded at: https://www.mdpi.com/article/10.3390/nu17010159/s1, Figure S1: Flow chart of the cohort study; Figure S2: Modifying effect of *MTHFR C677T* genotype on the restricted cubic spline of the dose–response relationship between serum folate concentrations and stroke.
