# Supplementary material for: Association Between Serum Folate Concentrations and 10-Year Stroke Risk in a Prospective Community Cohort: Mediation and Interaction Analyses
Source: Nutrients. 2024 Dec 31;17(1):159. doi: 10.3390/nu17010159 (PMC11722758; doi:10.3390/nu17010159)
Supplement: Supplementary file 1 [file nutrients-17-00159-s001.zip › nutrients-3345674-supplementary.pdf]

Association Between Serum Folate Concentrations and 10-Year Stroke Risk in a Prospective Community Cohort: Mediation and Interaction Analyses

Supplementary Materials

Figure S1 Flow chart of the cohort study

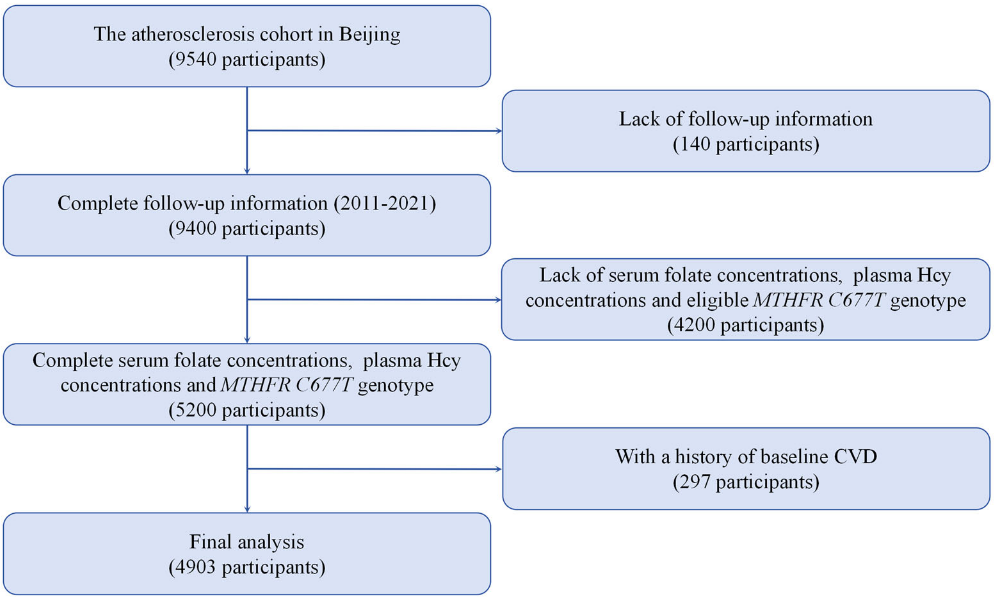

CVD: cardiovascular disease; Hcy: homocysteine; MTHFR: methylenetetrahydrofolate reductase.

Figure S2 Modifying effect of *MTHFR C677T* genotype on the restricted cubic spline of the dose-response relationship between serum folate concentrations and stroke

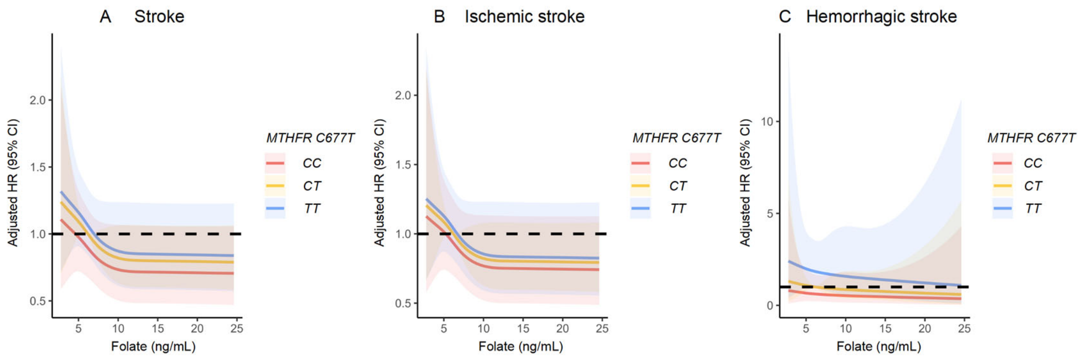

CI: confidence interval; HR: hazard ratio; MTHFR: methylenetetrahydrofolate reductase.

Restricted cubic spline was adjusted for baseline age, sex, BMI, eGFR, drinking, smoking, hypertension, dyslipidemia, diabetes, the use of antihypertensive, lipid-lowering, and hypoglycemic drugs, and plasma Hcy concentrations. The shaded regions of varying colors denote the 95% CI for the restricted cubic spline. The X-axis is bounded by the 0.5th and 99.5th percentiles of serum folate concentrations.
